# 3-β-Εrythrodiol isolated from *Conyza canadensis* inhibits MKN-45 human gastric cancer cell proliferation by inducing apoptosis, cell cycle arrest, DNA fragmentation, ROS generation and reduces tumor weight and volume in mouse xenograft model

**DOI:** 10.3892/or.2021.7980

**Published:** 2021-02-18

**Authors:** Kai Liu, Yue-Hong Qin, Jian-Yong Yu, Heng Ma, Xi-Lin Song

Oncol Rep 35: 2328-2338, 2016; DOI: 10.3892/or.2016.4610

Following the publication of the above paper, an interested reader drew to our attention that a number of apparent anomalies existed with the data presented in the above paper; specifically, there appeared to be strikingly similar and replicated patternings of the cells within the cellular images featured in Figs. 4A-D and 5A-D (affecting all the figure parts). Secondly, Fig. 4 in the above study appeared to have been a reproduction of Fig. 3 from following paper featuring different authors, albeit Fig. 4 appeared in greyscale, and not in colour, in the above paper [Wan G, Tao J-G, Wang G-D, Liu. S-P, Zhao H-X and Liang Q-D: *In vitro* antitumor activity of the ethyl acetate extract of *Potentilla chinensis* in osteosarcoma cancer cells. Mol Med Rep 14: 3634-3640, 2016].

Following an internal enquiry, the Editor of *Oncology Reports* was able to verify the claims made by the interested reader; therefore, in view of the number of potential anomalies that have been identified and owing to a lack of overall confidence in the presented data, the Editorial Board have decided to retract the above paper from the publication. The Journal were also contacted independently by the authors, who wished to retract the paper on account of not being able to reproduce the results shown in Fig.. 2. The Editor apologizes to the readership of the Journal for any inconvenience caused.

